# Effect of Ocular Dominance on Ocular Blood Flow Parameters

**DOI:** 10.7759/cureus.46500

**Published:** 2023-10-04

**Authors:** Yuta Nakaniida, Fumiko Higashikawa, Kana Tokumo, Yuki Yuasa, Hiromitsu Onoe, Naoki Okada, Shunsuke Nakakura, Ryo Asaoka, Yoshiaki Kiuchi

**Affiliations:** 1 Ophthalmology and Visual Science, Hiroshima University, Hiroshima, JPN; 2 Probiotic Science for Preventive Medicine, Hiroshima University, Hiroshima, JPN; 3 Ophthalmology and Visual Science, Hiroshima university, Hiroshima, JPN; 4 Ophthalmology, Tsukazaki Hospital, Himeji, JPN; 5 Ophthalmology, Seirei Hamamatsu General Hospital, Hamamatsu, JPN

**Keywords:** optic nerve head, blood flow velocity, laser speckle flowgraphy, ocular blood flow, ocular dominance

## Abstract

Purpose: In binocular vision, there is a dominant eye and a nondominant eye, a phenomenon termed ocular dominance. This study determined the differences and associations of the ocular blood flow parameters between dominant and nondominant eyes in healthy Japanese subjects.

Methods: This cross-sectional study included 128 eyes of 64 subjects (13 male and 51 female) aged ≥ 20 years. The ocular blood flow parameters were assessed using laser speckle flowgraphy (LSFG), and software was used to calculate the mean blur rate (MBR), which reflects the blood flow velocity.

Results: There were no significant differences in axial length (AL), spherical equivalent (SE), intraocular pressure (IOP), uncorrected visual acuity (UCVA), best-corrected visual acuity (BCVA), or ocular blood flow parameters between the dominant and nondominant eyes. The ocular blood flow parameters of the dominant eye were significantly and positively correlated with those of the nondominant eye (all P < 0.001).

Conclusions: No significant differences in ocular blood flow parameters exist between the dominant and nondominant eyes in healthy subjects. The ocular blood flow parameters in the dominant eye are associated with those in the nondominant eye.

## Introduction

Humans typically use both eyes to view objects. In binocular vision, one eye functions dominantly over the other eye, a phenomenon termed ocular dominance [1,2]. The accommodative response velocity is significantly faster and the accommodative response time is significantly shorter in dominant eyes than in nondominant eyes [3]. The P50 amplitude, which reflects the macular cone function in the pattern electroretinogram, is significantly increased in dominant eyes [4]. In addition, the axial length (AL) has been reported to be longer and the incidence of myopia is greater in the dominant eye [5,6]. These findings suggest that there are functional and structural differences between dominant and nondominant eyes.

Recently, laser speckle flowgraphy (LSFG) was developed to provide detailed information regarding ocular blood flow, and it has been widely used to examine ocular blood flow parameters [7]. Ocular blood flow parameters are associated with the development and progression of glaucoma [8,9]. Therefore, ocular blood flow parameters may related to visual function and affected by ocular dominance, visual functions, and visual structures.

This cross-sectional study aimed to determine the differences in ocular blood flow parameters between the dominant and non-dominant eyes of healthy Japanese subjects using LSFG. The associations of the ocular blood flow parameters in the dominant and nondominant eyes were also investigated.

This article was previously presented as a meeting abstract at the 14th Joint Meeting of Japan-China-Korea Ophthalmologists on November 27, 2021.

## Materials and methods

This study was approved by the Ethical Committee for Clinical Research at Hiroshima University (approval number C-329-1). Written informed consent was obtained from the patients for their information to be stored in the hospital database and used in this study. This study was conducted in accordance with the Declaration of Helsinki.

Subjects

This cross-sectional study included 128 eyes from 64 healthy Japanese subjects who were enrolled between September 2021 and December 2021. Subjects aged ≥ 20 years with a best-corrected visual acuity (BCVA) of ≥ 20/20 and no ocular diseases other than refractive error were included in this study. Subjects were also required to have no history of smoking, no history of refractive or intraocular surgery, a systolic blood pressure (SBP) < 160 mmHg, and a diastolic blood pressure (DBP) < 100 mmHg. The mean arterial blood pressure (MAP) and pulse pressure amplitude (PPA) were calculated in the seated position as MAP = SBP + 1/3 (SBP - DBP) and PPA = SBP - DBP.

Ocular dominance

The dominant eye was determined using the hole-in-card test [10]. During this test, a card with a 3-cm hole in the center is held at arm’s length, and the subjects view a 0.1 Landolt ring chart that is five meters away using both eyes. Then, the subjects close both eyes alternately. The eye that is viewing the object through the hole is determined to be the dominant eye. Each subjects performed the test three times. If the results of the three tests did not match, the participant was excluded from the study. However, no participant in this study was excluded based on this criterion.

Laser Specle Flowgraphy

Ocular blood flow was assessed using LSFG (Softcare Co., Ltd., Fukuoka, Japan). The subjects’ blood pressure and heart rate were measured after they sat in a quiet, dark room for five minutes.

The principle of LSFG has been previously described in detail [7]. The LSFG consists of a fundus camera equipped with a diode laser (830-nm wavelength) and an ordinary charge-coupled device camera (750 × 360 pixel resolution). A total of 118 images were continuously acquired in approximately four seconds at 30 frames/s, with an exposure time of 1/500 s. The accompanying analysis software (LSFG Analyzer, Version 3.2.3.0; Softcare Co., Ltd.) automatically detected the beginning and end of the recorded cardiac cycle within 4 s of the acquisition time. In this system, the laser light is scattered by the movement of red blood cells and the interference of the scattered light forms a speckled pattern. The mean blur rate (MBR) was obtained by calculating the temporal velocity change in the speckle pattern, which reflected the blood flow velocity. One experienced operator (Y N) set the band, termed the region of interest, by referring to fundus photographs to specify the area to be observed on the color map obtained from the analysis.

In this study, the “follow up scan” function of the LSFG Analyzer software was used to maintain the same band position and size in all subsequent scans of the same participant. An ellipsoid band was placed at the border of the optic nerve head (ONH), and the subjects fixated on the nasal internal optotype. The LSFG Analyzer software automatically divided an ellipsoid band into large vessels and tissue areas and calculated the MBR in each area. The ellipsoid band was automatically divided into four sectors: superior, temporal, inferior, and nasal. However, in this study, the four areas were totaled and analyzed as a whole. The MBRs in the vessel area (MV) and tissue area (MT) of the ONH were calculated. The MT is the sum of blood flow information from surface capillaries in areas other than the retinal vessels in the ONH to the lamina cribrosa [11].

To analyze the macular blood flow, a rectangular band (150 × 150 pixels) was placed at the fixation point (the macula) as the subjects fixated on the frontal internal optotype [12,13]. The MBR in the macular area of the choroid (CHOROID) reflects the blood flow velocity in this area, representing the choroidal circulation [12]. The mean value of three measurements was used for the analysis. While ocular blood flow parameters should ideally be adjusted for interindividual comparisons, no interindividual comparisons were made in this study, as the differences and associations between dominant and non-dominant eyes in individuals were investigated [14]. Therefore, no adjustments were made.

Other measurements

The AL was measured using IOL Master (Carl Zeiss Meditec, Dublin, USA). The intraocular pressure (IOP) was measured using a Goldman applanation tonometer. The uncorrected visual acuity (UCVA) and BCVA were measured using a Landolt ring chart at a distance of 5 m. The UCVA and BCVA were calculated as the logarithm of the minimum angle of resolution (logMAR) units.

Statistical analysis

Continuous variables are presented as mean ± standard deviation and range. Categorical variables are presented as numbers and percentages. The distribution of each variable was checked for normality using the Shapiro-Wilk test. None of the variables showed normal distribution. Therefore, nonparametric tests were performed.

The nonparametric Wilcoxon signed-rank test [15] was used to compare values obtained for dominant and nondominant eyes obtained from the same subjects. The associations of the ocular blood flow parameter (MV, MT, and CHOROID) between the dominant and nondominant eyes were determined using the nonparametric Spearman’s rank correlation coefficient.

Statistical significance was set at P = 0.05, and all tests were two-tailed. The Bonferroni correction was applied to the P value to account for multiple comparisons [16,17].

The statistical programming language R (R version 4.1.2; The Foundation for Statistical Computing, Vienna, Austria) was used for all statistical analyses.

## Results

Of the 64 subjects, 13 (20.4%) were male and 51 (79.6%) were female (Table 1). 40 (62.5%) subjects had a dominant right eye and 24 (37.5%) had a dominant left eye.

**Table 1:** Subjects’ demographics SBP: Systolic Blood Pressure; DBP: Diastolic Blood Pressure; MAP: Mean Arterial Blood Pressure; PPA: Pulse Pressure Amplitude

Parameters	Mean ± SD	Range
Subjects	64	-
Eyes	128	-
Age (years)	51.08 ± 10.35	20.00 to 71.00
Gender (male/female)	13 / 51	-
Dominant eye (R/L)	40 /24	-
Height (cm)	159.35 ± 6.51	148.60 to 175.20
Weight (kg)	54.47 ± 9.94	35.80 to 79.00
BMI (kg/m^2^)	21.35 ± 2.96	15.00 to 28.00
SBP (mmHg)	106.45 ± 16.97	76.00 to 159.50
DBP (mmHg)	62.68 ± 12.03	32.50 to 95.50
MAP (mmHg)	121.03 ± 19.08	90.50 to 182.50
PPA (mmHg)	43.77 ± 8.03	29.00 to 69.00
Heart Rate (bpm)	75.59 ± 11.53	53.00 to 103.00

There were no significant differences in AL, spherical equivalent (SE), IOP, UCVA, BCVA, MV, MT, or CHOROID between the dominant and nondominant eyes (Table 2).

**Table 2:** Comparison of the dominant and nondominant eyes AL: Axial Length; SE: Spherical Equivalent; IOP: Intraocular Pressure; UCVA: Uncorrected Visual Acuity; BCVA: Best-Corrected Visual Acuity; logMAR: Logarithm of the Minimum Angle of Resolution; MV: Mean Blur Rate in the Vessel Area; MT: Mean Blur Rate in the Tissue Area; CHOROID: Mean Blur Rate in the Choroid at the Aacular Area; AU: Arbitrary Units P < 0.05, Wilcoxon signed-rank test P value were adjusted by Bonferroni correction.

Parameters	Dominant eye			Non-dominant eye		P value	Adjusted P value
	Mean ± SD	Range		Mean ± SD	Range		
AL (mm)	24.64 ± 1.35	21.49 to 27.29		24.61 ± 1.41	21.54 to 28.21	0.47	1.00
SE (diopters)	-2.57 ± 2.69	-8.75 to 1.88		-2.56 ± 2.78	-9.63 to 2.50	0.98	1.00
IOP (mmHg)	11.83 ± 2.61	7.00 to 20.00		11.84 ± 2.37	8.00 to 19.00	0.90	1.00
UCVA (logMAR)	0.51 ± 0.53	-0.30 to 1.52		0.49 ± 0.54	-0.18 to 1.40	0.55	1.00
BCVA (logMAR)	-0.16 ± 0.08	-0.30 to 0.00		-0.16 ± 0.07	-0.30 to 0.00	0.65	1.00
MV (AU)	47.88 ± 7.62	29.60 to 65.70		46.77 ± 6.65	23.90 to 60.90	0.09	0.72
MT (AU)	12.44 ± 2.58	7.10 to 18.40		12.30 ± 2.48	8.50 to 19.10	0.23	1.00
CHOROID (AU)	10.15 ± 3.98	4.00 to 20.80		9.49 ± 3.96	3.80 to 20.10	0.10	0.80

**Figure 1:**
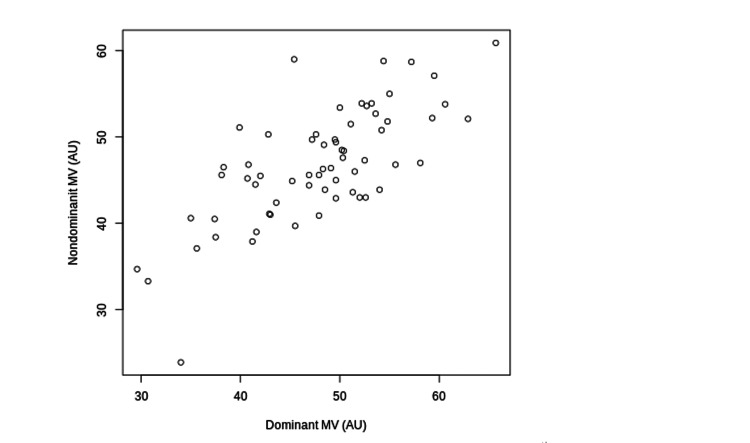
The Correlations between MV of the Dominant and Non-Dominant Eyes The correlations between MV of the dominant and nondominant eyes are shown. MV: Mean Blur Rate in the Vessel Area; AU: Arbitrary Units

**Figure 2:**
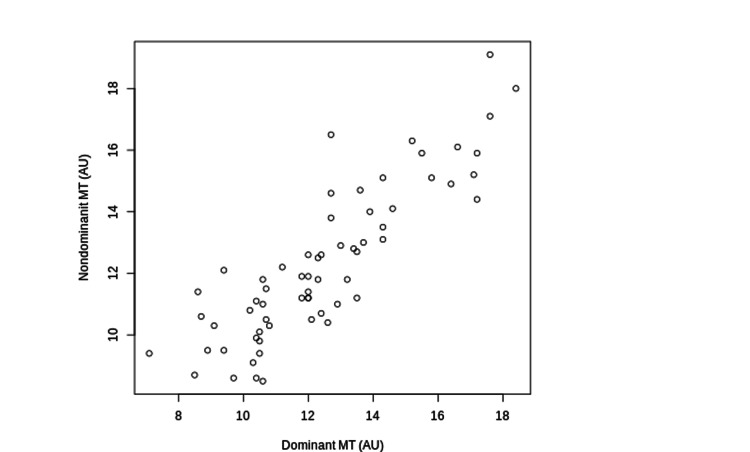
The Correlations between MT of the Dominant and Non-Dominant Eyes The correlations between MT of the dominant and nondominant eyes are shown. MT: Mean Blur Rate in the Tissue Areas: AU: Arbitrary Units

**Figure 3:**
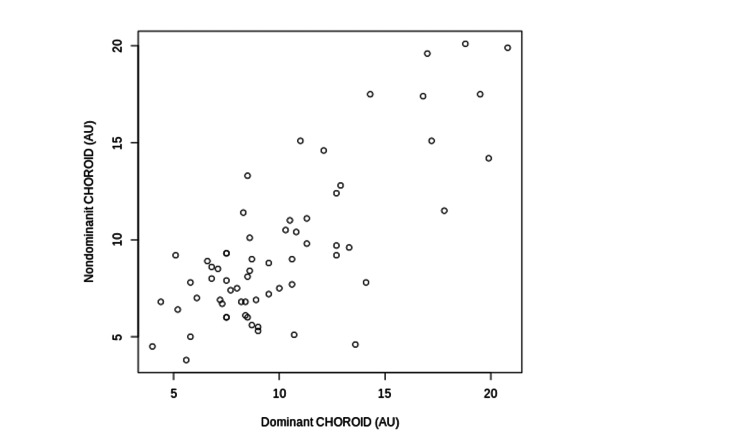
The Correlations between CHOROID of the Dominant and Non-Dominant Eyes The correlations between CHOROID of the dominant and nondominant eyes are shown. CHOROID: Mean Blur Rate in the Macular Area of the Choroid; AU: Arbitrary Units

MV (r = 0.68, P < 0.001), MT (r = 0.85, P < 0.001), and CHOROID (r = 0.62, P < 0.001) of the dominant and nondominant eyes were significantly correlated (Figures 1-3).

## Discussion

The ocular blood flow parameters were not different between the dominant and nondominant eyes in this study. The ocular blood flow parameters of the dominant eye were significantly and positively correlated with those of the nondominant eye.

Other instruments to measure ocular blood flow

In addition to LSFG, several other instruments measure ocular blood flow, including fluorescein angiography and indocyanine green angiography [18,19]. Although these testing methods allow for the quantitative evaluation of blood flow, it is difficult to compare blood flow velocities between the left and right eyes using these methods. Color Doppler imaging is used to measure ocular arterial blood flow velocity, and laser Doppler velocimetry is used to measure retinal blood flow based on the blood flow velocity and vessel diameter [20,21]. Unlike laser Doppler velocimetry, laser Doppler flowmetry measures blood flow in the choroidal and optic papillary capillaries, and optical coherence tomography angiography provides several images of the same area, allowing for the extraction of the moving signal to create a vascular image and a quantitative evaluation [22,23].

Clinical implications

Ocular blood flow parameters were not significantly different between the dominant and nondominant eyes in this study and showed a significant positive correlation. It was found that ocular dominance had little effect on ocular blood flow in healthy subjects. Furthermore, there were no significant differences in AL, SE, IOP, UCVA, and BCVA. Ocular dominance was also found to have little effect on ocular structure, visual acuity, and IOP in healthy subjects. Previous reports have reported no significant differences in contrast sensitivity, retinal sensitivity, reading speed, regulation, and fixation variability between the dominant and nondominant eyes in healthy subjects [24-28]. These previous reports suggest that ocular dominance has little effect on visual function in healthy subjects. ocular blood flow supplies oxygen and nutrients to the eye. it makes sense that there would be no significant difference in ocular blood flow and no significant difference in visual function. Since the left-right balance is often maintained in healthy subjects, ocular dominance is not considered to have a significant effect.

Interpretation of correlations

In this study, ocular blood flow all showed positive correlations; MT showed stronger correlations than MV. This may be due to the fact that MT has a larger area and more information at the time of analysis. This suggests that MT is more appropriate than MV for evaluating blood flow in the ONH. In addition, MT showed a stronger correlation than CHOROID in this study. This suggests that blood flow in the ONH tissue area is more closely related to the dominant and nondominant eyes than blood flow in the choroid of the macula. 

Study limitations

This study has several limitations. First, the majority of subjects in this study (80%) were female; ocular blood flow has been reported to be significantly higher in females [29]. Second, most of the subjects were middle-aged and older, with a few younger subjects; MV and MT decrease with age [14,30].

Future directions

Future studies are needed to investigate subjects with left-right differences in refractive values, such as patients with anisometropia and subjects with a dominant eye with worse visual acuity and a nondominant eye with better visual acuity.

## Conclusions

In conclusion, the ocular blood flow parameters are not significantly different between the dominant and nondominant eyes in healthy Japanese subjects. There is a strong, positive association between the ocular blood flow in the dominant and nondominant eyes. In particular, blood flow in the tissue area of the ONH showed a stronger correlation than blood flow in the vessel area of the ONH and blood flow in the macular area of the choroid.
